# Selective Targeting of Proteins by Hybrid Polyoxometalates: Interaction Between a Bis-Biotinylated Hybrid Conjugate and Avidin

**DOI:** 10.3389/fchem.2018.00278

**Published:** 2018-07-11

**Authors:** Valeria A. Zamolo, Gloria Modugno, Elisa Lubian, Alessandro Cazzolaro, Fabrizio Mancin, Livia Giotta, Disma Mastrogiacomo, Ludovico Valli, Alessandra Saccani, Silke Krol, Marcella Bonchio, Mauro Carraro

**Affiliations:** ^1^Department of Chemical Sciences, University of Padova and ITM-CNR, Padova, Italy; ^2^Department of Biological and Environmental Sciences and Technologies – DiSTeBA, University of Salento, Lecce, Italy; ^3^NanoMed Lab, Fondazione IRCCS Institute of Neurology “Carlo Besta,” Milan, Italy; ^4^Laboratory of Translational Nanotechnology, IRCCS Oncologic Institute “Giovanni Paolo II,” Bari, Italy

**Keywords:** biotin, avidin, polyoxometalates, recognition, surface plasmon resonance, bio-hybrids, oxidation catalysis

## Abstract

The Keggin-type polyoxometalate [γ-SiW_10_O_36_]^8−^ was covalently modified to obtain a bis-biotinylated conjugate able to bind avidin. Spectroscopic studies such as UV-vis, fluorimetry, circular dichroism, coupled to surface plasmon resonance technique were used to highlight the unique interplay of supramolecular interactions between the homotetrameric protein and the bis-functionalized polyanion. In particular, the dual recognition mechanism of the avidin encompasses (i) a complementary electrostatic association between the anionic surface of the polyoxotungstate and each positively charged avidin subunit and (ii) specific host-guest interactions between each biotinylated arm and a corresponding pocket on the tetramer subunits. The assembly exhibits peroxidase-like reactivity and it was used in aqueous solution for L-methionine methyl ester oxidation by H_2_O_2_. The recognition phenomenon was then exploited for the preparation of layer-by-layer films, whose structural evolution was monitored *in situ* by ATR-FTIR spectroscopy. Finally, cell tracking studies were performed by exploiting the specific interactions with a labeled streptavidin.

## Introduction

The preparation of bio-inorganic conjugates is currently investigated for the preparation of biosensors, metal-based antibiotics, radiopharmaceuticals, anti-cancer drugs, and imaging contrast agents (Orvig and Abrams, 1999; Barry and Sadler, 2013; Albada and Metzler-Nolte, 2016; Liu et al., 2016). In addition, bio-hybrid nanostructures are emerging as innovative functional materials (Wortmann et al., 2014; Liu et al., 2016). Among inorganic nanodrug candidates, polyoxometalates (POMs) are multi- metallic and polyanionic oxides which have shown interesting potential applications as antibacterial, antiviral, antitumoral agents (Rhule et al., 1998; Hasenknopf, 2005; Bijelic et al., 2018). Such biological activity mainly derives from their redox behavior, their biomimetic activity, or from their capability to interact with biological macromolecules through electrostatic interactions (Prudent et al., 2008; Li et al., 2016). Due to their nanosized dimension and polyanionic charge, indeed, POMs can easily interact with positively charged domains of peptides and proteins, affecting their secondary/tertiary structure and altering their functionalities (Wu et al., 2005; Zhang et al., 2008; Geng et al., 2011). On the other hand, the competition with electron rich natural substrates, such as DNA and ATP, can also lead to the inhibition of enzymatic processes (Judd et al., 2001; Prudent et al., 2010; Iqbal et al., 2013; Stephan et al., 2013). To control this behavior, POMs can be engineered to tune their polarity, redox potential, shape, acidity and surface charge distribution (Rhule et al., 1998; Hasenknopf, 2005; Bijelic et al., 2018). However, since inorganic POMs present low hydrolytic stability at physiologically relevant pH values, leading to cytotoxic derivatives, many efforts have been made to modify their structure and composition, in order to obtain compounds with low toxicity, higher stability and selectivity (Wang et al., 2003). In particular, the covalent functionalization of POMs with organic pendants (Dolbecq et al., 2010; Proust et al., 2012) imparts higher stability under physiological conditions, and offers an appealing strategy for improving their bio-distribution (Dong et al., 2011; Flütsch et al., 2011; Yang et al., 2013; Fu et al., 2015; Karimian et al., 2017; Linnenberg et al., 2017). There is a definite potential of hybrid, organic-inorganic, POMs to trigger the recognition of cellular receptors and of biological matter, although with few cases (Li et al., 2013; Ventura et al., 2018). In this direction, we have designed a tweezer-like (Carraro et al., 2012a; Modugno et al., 2014) biotinylated POM (Prudent et al., 2008; Linnenberg et al., 2017) in order to exploit the well-known avidin-biotin complex (ABC).

The affinity between biotin (vitamin H) and the homotetrameric avidin is known as one of the strongest non-covalent interactions in nature, with a dissociation constant K_D_ = 10^−15^ M. Avidin is the natural transport protein of biotin and the biotin-avidin association is routinely exploited in several biochemical assays. In addition, since avidin can expose a diffuse positive charge (with an isoelectric point, pI, of about 10.5), the complementary electrostatic interaction with the negative POM surface can be a further assembly drive. We show herein that a bis-functionalized decatungstosilicate complex with formula (^*n*^Bu_4_N)_3_H[γ-SiW_10_O_36_{(C_5_H_7_N_2_OS)(CH_2_)_4_CONH(CH_2_)_3_Si}_2_O] (**TBA-POM-biot**_2_) interacts with the avidin target by an interplay of electrostatic and host-guest binding interactions, which set the basis for novel supramolecular bio-conjugates with applications in drug delivery, catalysis and material sciences. In particular, surface plasmon resonance (SPR), UV-vis, circular dichroism (CD), fluorescence spectroscopy and attenuated total reflectance Fourier transform infrared spectroscopy (ATR-FTIR) evidences are compared and contrasted vis-à-vis the association properties of biotin-free POMs, as well as considering the stoichiometry/geometry of the resulting bio-hybrid adduct.

Our results include catalytic tests in the presence of H_2_O_2_ as co-factor, which highlight the functional response of the POM surface as artificial peroxidase, and a preliminary investigation on cell internalization (Dong et al., 2011; Flütsch et al., 2011; Yang et al., 2013; Fu et al., 2015; Karimian et al., 2017; Linnenberg et al., 2017) of the biotinylated POM by means of labeled streptavidin.

## Experimental section

K_8_[γ-SiW_10_O_36_] (**K-POM**), (Canny et al., 1986) (^*n*^Bu_4_N)_4_[γ-SiW_10_O_34_(H_2_O)_2_] **TBA-POM** (Kamata et al., 2003) (^*n*^Bu_4_N)_4_[γ-SiW_10_O_36_**{**NH_2_(CH_2_)_3_Si**}**_2_O] (**TBA-POM-NH**_2_) (Carraro et al., 2006, 2012a; Modugno et al., 2014) were prepared as described in the literature.

Phosphate saline buffer (PBS) was prepared dissolving sodium phosphate 0.01 M, sodium chloride 0.14 M, potassium chloride 0.03 M in deionized water and used in all experiments.

**Synthesis of (**^*n*^**Bu**_4_**N)**_3_**H[**γ**-SiW**_10_**O**_36_**{(C**_5_**H**_7_**N**_2_**OS)(CH**_2_**)**_4_**CONH(CH**_2_**)**_3_**Si}**_2_**O]** (**TBA-POM-biot**_2_): Biotin (37 mg, 151 μm) was introduced in a well dried Schlenk with magnetic stirring, under N_2_ atmosphere. Anhydrous DMF (0.5 mL) and CH_3_CN (1 mL), N,N′-dicyclohexylcarbodiimide DCC (33 mg, 160 μm) and N*-*hydroxysuccinimide NHS (19 mg, 164 μm) were then added. The reaction mixture, vigorously stirred, was allowed to react for one night at 50°C, under nitrogen. Then, **TBA-POM-NH**_2_ (200 mg, 59.4 μm) and TEA (21.3 μL, 149 μm) were dissolved in 2 ml of anhydrous CH3CN and added to the reaction mixture. The mixture was stirred for 1 day at room temperature. Finally, the reaction mixture was centrifuged to remove insoluble reagents and byproducts. The volume of the solution was reduced to 1 mL, upon evaporation under vacuum, then water was added to precipitate the product. The solid was washed with water (3 times) and diethyl ether (3 times) on a fritted funnel under vacuum. 154 mg of product were obtained (68% yield).

FT-IR (KBr, cm^−1^): 2961 (m), 2,934 (m), 2,873 (m), 1,662 (m), 1,469 (m), 1,387 (w), 1,099 (w), 948 (m), 901 (s), 820 (s), 734 (s), 544 (w); ^1^H NMR (300 MHz, CD_3_CN, δ): 0.55 (4 H, m), 0.99 (48 H, m), 1.39 (32 H, m), 1.64 (32 H, m), 2.63 (2 H, m), 3.15 (32 H, m), 4.33 (2 H, m), 4.49 (2 H, m), 5.09 (2 H, s, br), 5.85 (2H, s, br), 6.89 (2 H, s, br); ^13^C NMR (75.5 MHz, CD_3_CN, 301 K, δ): 14.08 (32 C), 20.42 (32 C), 21.96 (2 C), 24.47 (32 C), 25.92 (2 C), 26.67 (2 C), 29.21 (2 C), 36.77 (2 C), 41.89 (2 C), 42.90 (2 C), 56.56 (2 C), 61.24 (2 C), 62.77 (2 C), 164.69 (2 C), 174.18 (2 C); ^29^Si NMR (CH_3_CN/CD_3_CN, 301 K, δ): −62.01 (2 Si, s), −88.43 (1 Si, s); ^183^W NMR (16.67 MHz, CH_3_CN/CD_3_CN, 301 K, δ): −107 .55 (4 W, s), −136.09 (2W, s), −142.08 (4 W, s); ESI-MS (-), CH_3_CN, *m/z:* calcd for [C_26_H_44_N_6_O_41_S_2_Si_3_W_10_]^4−^ 770.9; found, 768.2, Anal. calcd. for C_74_H_153_N_9_O_41_S_2_Si_3_W_10_ C 23.3; H 4.1; N 3.3; S 1.7; found: C 23.1; H 4.2; N 2.7; S: 0.9.

Synthesis of hybrid POMs as sodium salts: **Na**_4_**[**γ**-SiW**_10_**O**_36_**{(C**_5_**H**_7_**N**_2_**OS)(CH**_2_**)**_4_**CONH(CH**_2_**)**_3_**Si}**_2_**O] (Na-POM-biot**_2_**)** and **Na**_4_**[**γ**-SiW**_10_**O**_36_**{NH**_2_**(CH**_2_**)**_3_**Si}**_2_**O] (Na-POM-NH**_2_**)**: In around-bottomed flask, 100 mg of **TBA-POM-Biot**_2_ or **TBA-POM-NH**_2_ (24.7 μmol) were dissolved in 3 ml of acetonitrile. Then 26.7 mg of tetramethylammonium bromide (173 μmol), dissolved in 2 ml of water, were added. The reaction mixture was stirred at room temperature for one night. The solution obtained was then poured into EtOH (15 mL). The white precipitate obtained was filtered, dried under vacuum and, finally, eluted in a chromatography column (3 cm diameter, 40 cm length) partially filled (ca. 100 cm^3^ volume) with a cation exchange resin (Amberlyst 15) pre-loaded with sodium ions (1M NaCl overnight), using ca. 50 mL of water/acetonitrile mixtures with variable composition (from 50:50 to 100:0) as eluent. Finally, the solution was lyophilized to remove water. The Na-POMs were collected with ca. 40% yield. FT-IR of **Na-POM-biot**_2_ (KBr, cm^−1^): 3,464 (s,b), 2,928 (w), 2,870 (w), 1,684 (s), 1,635 (s), 1,558 (m), 1,541 (m), 1,458 (s), 1,270 (s), 1,039 (m), 958 (m), 883 (s), 824 (m), 753 (s), 528 (w). FT-IR of **Na-POM-NH**_2_ (KBr, cm^−1^): 997 (w), 862 (m), 901 (s), 797 (s), 744 (m), 517 (m).

### SPR measurements

SPR analysis was performed on a BIACORE 100 system. CM5 chips from BIACORE (Uppsala, Sweden) were used for all the experiments. Avidin was anchored to the chip via EDC-NHS activation of the surface. To this aim, a dextrane-coated gold chip (CM5) was activated by flowing a 1:1 mixture of 0.2 M N-ethyl-N-(3-dimethylaminopropyl) carbodiimide (EDC) and 0.05 M N-hydroxysuccinimide (NHS) in water. Avidin (50 μg/mL) in 10 mM sodium acetate (pH 5) was immobilized on the activated chip surfaces at a flow rate of 10 μL/min. Excess of activated carboxylic groups on the chip was blocked with ethanolamine. HBS-EP buffer (0.01 M HEPES pH 7.4, 0.15 M NaCl, 3 mM EDTA, 0.005% v/v Surfactant P20) was used as running buffer to dilute avidin and water soluble POM solutions and for avidin immobilization. HBS-EP buffer with 5% DMSO was used to dilute all the other POMs solution and as running buffer in the corresponding experiments. All the solutions were filtered on a 0.22 μm membrane prior to use. All the experiments were conducted at 25°C with constant flux of 10 μl/min. Association and dissociation phases were 200 s and 100 s long, respectively. After each experiment, the surface was regenerated using 1 M NaCl in 50 mM NaOH. The recovery of the initial RU count was controlled before considering chip reutilization. The kinetic parameters were calculated using the BIACORE evaluation software on a personal computer. Analysis and fitting were performed using the bridging ligand model.

### ATR-FTIR monitoring of LBL self-assembly

ATR-FTIR spectra were acquired with a Perkin Elmer Spectrum One spectrometer equipped with an ATR horizontal sampling apparatus. The internal reflection element (IRE) was a three bounce 4 mm diameter diamond microprism (Smith Detection, USA, former SensIR technologies). The spectral resolution used for all measurements was 4 cm^−1^. Before each experiment, the diamond crystal was polished with an aqueous 0.05 μm Al_2_O_3_ slurry and then rinsed with deionized water and ethanol. The deposition of alternate layers of **Na-POM-biot**_2_ and avidin onto the diamond surface was achieved in a flow-based “layer by layer” manner by means of a cylindrical flow cell clamped onto the ATR plate and sealed via a Parafilm gasket, with an internal volume of 150μL. Spectra were acquired while **Na-POM-biot**_2_ or avidin solutions were flowed across the surface of the IRE at a flow rate of 2.2 mL min^−1^ using a peristaltic pump. Both POM and avidin were dissolved in PBS buffer at pH 7.0. Final POM and avidin concentrations were 0.13 and 0.4 g/L (40 and 6.3 μM) respectively. *A positively charged protein as cytochrome c (pI* = *10.0–10.5, close to that of avidin) can be easily bound to* ***Na-POM-biot***_2_ *layers, while a negatively charged protein as GOx (pI* = *4.05) suffers from a strong repulsion with the polyanion, which is completely rinsed from the surface*.

### Cell culture and imaging

Human cervical carcinoma cells (HeLa) were grown in a standard culture media at 37°C and in 5% CO_2_ atmosphere. Cells were seeded in a μ-Slide 8-well ibidi plate (Martinsried, Germany) at a density of 5 × 10^4^ cells per well (1.0 cm^2^) and were allowed to adhere overnight. Before cell incubation with POMs, the medium was changed. Cells were then incubated with 0.2 or 0.4 mg/ml of **Na-POM-NH**_2_ or **Na-POM-biot**_2_ for 24 h at 37°C. After incubation the cells were washed 3 times with PBS, fixed with paraformaldehyde, permeabilized by Triton and then stained with Atto 633-Streptavidin (Sigma #00336). Nuclei were counterstained with Hoechst 33342 (Invitrogen, Oregon, USA), according to manufacturer's instructions. Cellular uptake and internalization of biotin labeled POMs was visualized and evaluated with an inverted confocal laser scanning microscope (CLSM; Carl Zeiss LSM 510) equipped with a 63 × /1.3 oil DIC objective, using excitation lines at 405 (Hoechst 33342) and 633 nm (Atto 633-Streptavidin 638/658). ImageJ software was used for image analysis. PI staining and FACS (Fluorescence-activated cell sorting) signals were analyzed accordingly to a literature protocol (Riccardi and Nicoletti, 2006).

## Results and discussion

### Synthesis

The bis-biotinylated POM has been obtained starting from the divacant decatungstosilicate K_8_[γ-SiW_10_O_36_] K-POM, which has been initially treated with aminopropyl triethoxysilane (APTES) and tetrabutylammonium bromide to yield the doubly functionalized amino-derivative (^*n*^Bu_4_N)_3_H[γ-SiW_10_O_36_{NH_2_(CH_2_)_3_Si}_2_O] TBA-POM-NH_2_ (Scheme S1) (Carraro et al., 2006).

**Figure 1:**
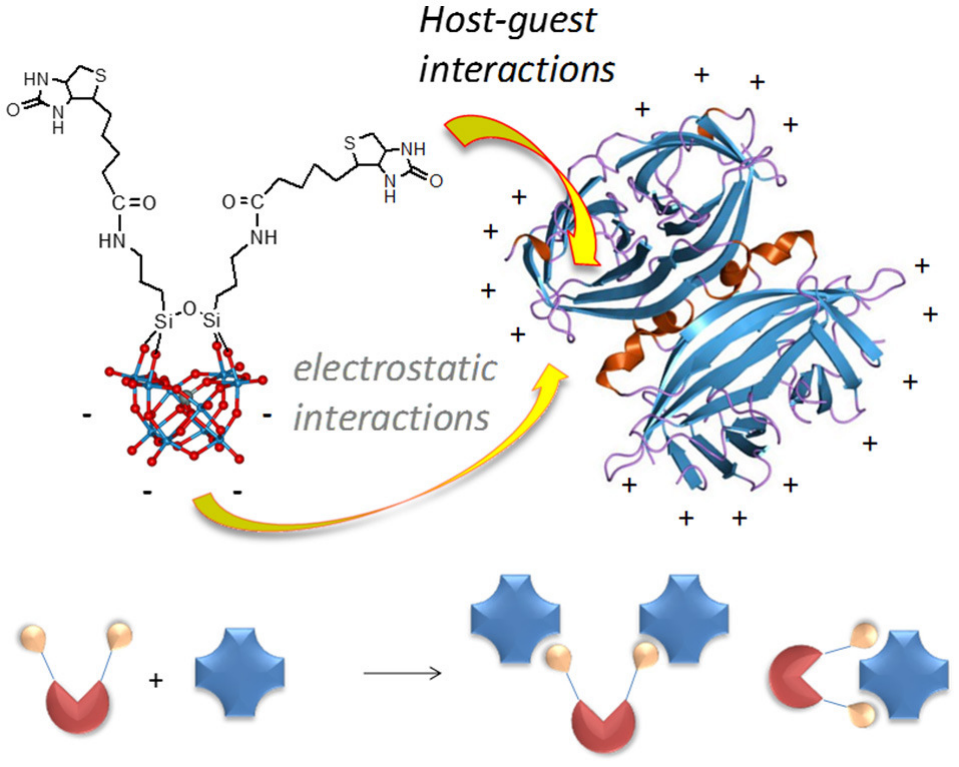
Structure of **POM-biot**_2_ [γ-SiW_10_O_36_{(C_5_H_7_N_2_OS)(CH_2_)_4_CONH(CH_2_)_3_Si}_2_O]^4−^ showing the organic domain, including the biotin and an aminopropyl spacer, and the ball & stick model of the inorganic scaffold (blue balls = W atoms; red balls = oxygen atoms), and possible POM binding modes (bridging or tweezer) with the avidin homotetramer.

The addition of dicyclohexylcarbodiimide (DCC) to a CH_3_CN/DMF solution of **POM-NH**_2_, biotin and N,N-diisopropylethylamine at 0°C, results in the formation of the biotinylated conjugate (^*n*^Bu_4_N)_3_H[γ-SiW_10_O_36_{(C_5_H_7_N_2_OS)(CH_2_)_4_CONH(CH_2_)_3_Si}_2_O] (**TBA-POM-biot**_2_) (68% yield, Figure 1). **TBA-POM-biot**_2_ has been characterized by ^1^H, ^13^C, ^29^Si, ^183^W NMR **(**CD_3_CN**)**, FT-IR, ESI-MS (Figures S1–S6) and elemental analysis. ^1^H and ^13^C NMR signals confirm the presence of biotin signals (among diagnostic peaks, three broad N*H*CO signals at 5.1, 5.9, 6.9 ppm and the two *C* = O signals at 164.7 and 174.2 ppm, Figures S1, S2), while heteronuclear (^29^Si and ^183^W, Figures S3, S4) NMR yield the typical signal patterns expected for a divacant Keggin structure decorated with a R-Si-O-Si-R tweezer-like motif,^11^ thus confirming the integrity of the POM scaffold after the post-functionalization with biotin. ESI-MS (negative mode, CH_3_CN, Figure S5) shows a peak at *m*/*z* = 768.2, due to the tetra-anionic species ([C_26_H_44_N_6_O_41_S_2_Si_3_W_10_]^4−^, calcd. *m*/*z* = 770.9). The corresponding water soluble salt (**Na-POM-biot**_2_**)** was obtained by replacing TBA counterions with Na^+^ on an ion-exchange resin (Figure S7).

### Study of the avidin/POM-Biot_2_ host-guest binding interaction

The interaction between avidin and **TBA-POM-biot**_2_ was investigated including a direct comparison with the biotin free precursors **TBA-POM-NH**_2_ (Carraro et al., 2006, 2012a; Modugno et al., 2014) and (^*n*^Bu_4_N)_4_[γ-SiW_10_O_34_(H_2_O)_2_] **TBA-POM**, (Kamata et al., 2003) their corresponding alkali metal salts **Na-POM-biot**_2_**, Na-POM-NH**_2_**, K-POM**, as well as the POM-free biotin (**Biot**). This approach is aimed at dissecting the diverse contribution of the POM components (anionic charge, satellite counterions, organic spacer and biotin-tweezer) that could play a role with respect to the avidin binding.

A first investigation on the interaction between avidin and **TBA-POM-biot**_2_ was performed by monitoring circular dichroism (CD, Figures S8–S11). Avidin, in the region 220–235 nm, shows a positive Cotton effect (λ_max_ = 227 nm), (Verdoliva et al., 2010) which is only slightly affected (15% decrease) by **Biot** guest, with a maximum decrease after addition of 4 guest equivalents (Figure S8). Addition of POMs has a stronger impact, with an abatement of the dichroic signal ranging from 50% (for **K-POM**) to 60% (for **POM-Biot**_2_), after addition of about one POM equivalent per avidin subunit. This evidence suggests that the POM scaffold by itself induces a modification of the protein structure, likely ascribed to electrostatic or hydrogen bond interactions between the inorganic POM surface and the protein residues Wu et al., 2005; Zhang et al., 2008; Geng et al., 2011; Li et al., 2013; Ventura et al., 2018).

**Figure 2:**
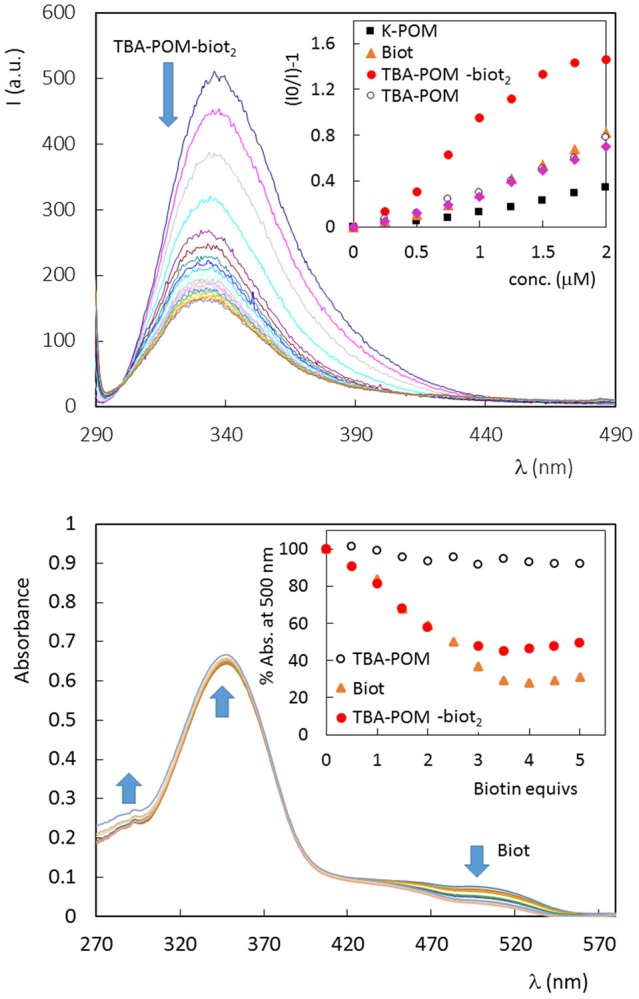
**(Top)** Fluorescence spectrum of avidin (1 × 10^−6^ M, in PBS 5% DMSO, 25°C) in the presence of increasing amount of **TBA-POM-biot**_2_ (0–6 μm) dissolved in PBS 5% DMSO. Excitation wavelength: 280 nm; 1 cm path length quartz cell. Inset: Stern-Volmer graphs, obtained for the fluorescence emission at 338 nm, in the presence of different POMs or **Biot**, in the range 0–2 μM. **(Bottom)** UV-vis spectra of tetrameric avidin-HABA (0.65 × 10^−6^ M, in HEPES buffer, 25°C) in the presence of increasing amount of biotin equivalents. Inset: % absorbance decrease observed at 500 nm in the presence of **Biot** (0–0.81 μM) or different POMs (0–0.41 μM, 1 biotin equiv. corresponding to 0.5 POM equivs.).

Fluorescence quenching is generally used to monitor the ABC host-guest interaction, that leads to the rearrangement of two or three tryptophan (Trp) residues shifting into an internal, more hydrophobic protein environment. These phenomena are responsible for a typical blue-shifted fluorescence quenching (Kurzban et al., 1989). This is indeed the case of avidin titrated with **Biot** or **TBA-POM-biot**_2_ (Figure 2, Figures S12, S13), which both give a partial quenching of the emission, with a wavelength shift from 338 to 331 nm. Monitoring of the fluorescence intensity ratio, (I_0_/I)-1, at 338 nm upon addition of **TBA-POM-biot**_2_(Figure 2, Figure S13), yields an initially upward curvature, suggesting the involvement of both static and dynamic quenching, followed by a different regime after the addition of 1 biotin equivalent per avidin subunit (corresponding to two POM equivalents per tetrameric avidin, 1 × 10^−6^ M). The lower slope of the second region is in agreement with a decreased affinity for additional POM units.

No fluorescence shift is observed with the biotin-free POMs (Figures S14–S16), that are instead responsible for a continuous static quenching, as reported in literature for other proteins, with Stern-Volmer constant K_SV_ = 10^4^-10^6^ M^−1^ (Zhang et al., 2007; Goovaerts et al., 2013). From the initial points of the Stern-Volmer plots (Figure 2, Figures S12–S16), it is indeed possible to compare the quenching efficiency of all samples, being >3 times higher for **TBA-POM-biot**_2_ (K_sv_ = 9.6 × 10^5^ M^−1^) than those obtained for the other samples (K_sv_ in the range 1–3 × 10^5^ M^−1^). This result highlights the dual role of both the biotin pendant and of the POM scaffold interacting with avidin.

To gain further insight on the binding nature, the affinity of all synthesized POMs toward avidin was investigated via UV-vis by means of the HABA (4′-hydroxyazobenzene-2-carboxylic acid, a molecule with lower affinity for avidin with respect to biotin) competitive titration probe (Figure 2, Figures S17–S20) (Skander et al., 2004). When the commercially available HABA-avidin adduct (K_D_ (HABA-avidin) = 10^−6^ M) is titrated with biotin, a progressive decrease of the 500 nm absorption peak, due to bound HABA, is observed. In this way, the number of the effective hosting sites can be monitored from the number of released HABA equivalents. Four biotin equivs are required to displace all HABA molecules. In our case, 2 equivalents of **TBA-POM-biot**_2_ are indeed enough to replace HABA, thus confirming the retention of the guest-specificity of both biotins installed on POM surface. As expected, the biotin-free POMs have no effect on HABA displacement (Figure 2, Figures S19, S20).

Concerning the binding geometry, **POM-biot**_2_ can adopt two possible arrangements: either one avidin serves as a di-topic receptor for the **POM-biot**_2_ tweezer, or **POM-biot**_2_ bridges two distinct avidins (Figure 1). Considering the steric hindrance of the POM scaffold, the latter binding mode is the most likely (Green et al., 1971; Geninatti Crich et al., 2005). The POM-bridging model was further inspected through a modified HABA-substitution titration. HABA-avidin was first titrated with 3 equivalents of biotin, in order to generally leave only one binding site occupied by HABA per protein (Figure S21). When 0.5 equivalents of **TBA-POM-biot**_2_ were added to the solution, they displaced all the remaining avidin-bound HABA, suggesting that each conjugate may easily arrange in a bridging conformation where two avidins are simultaneously bound.

**Figure 3:**
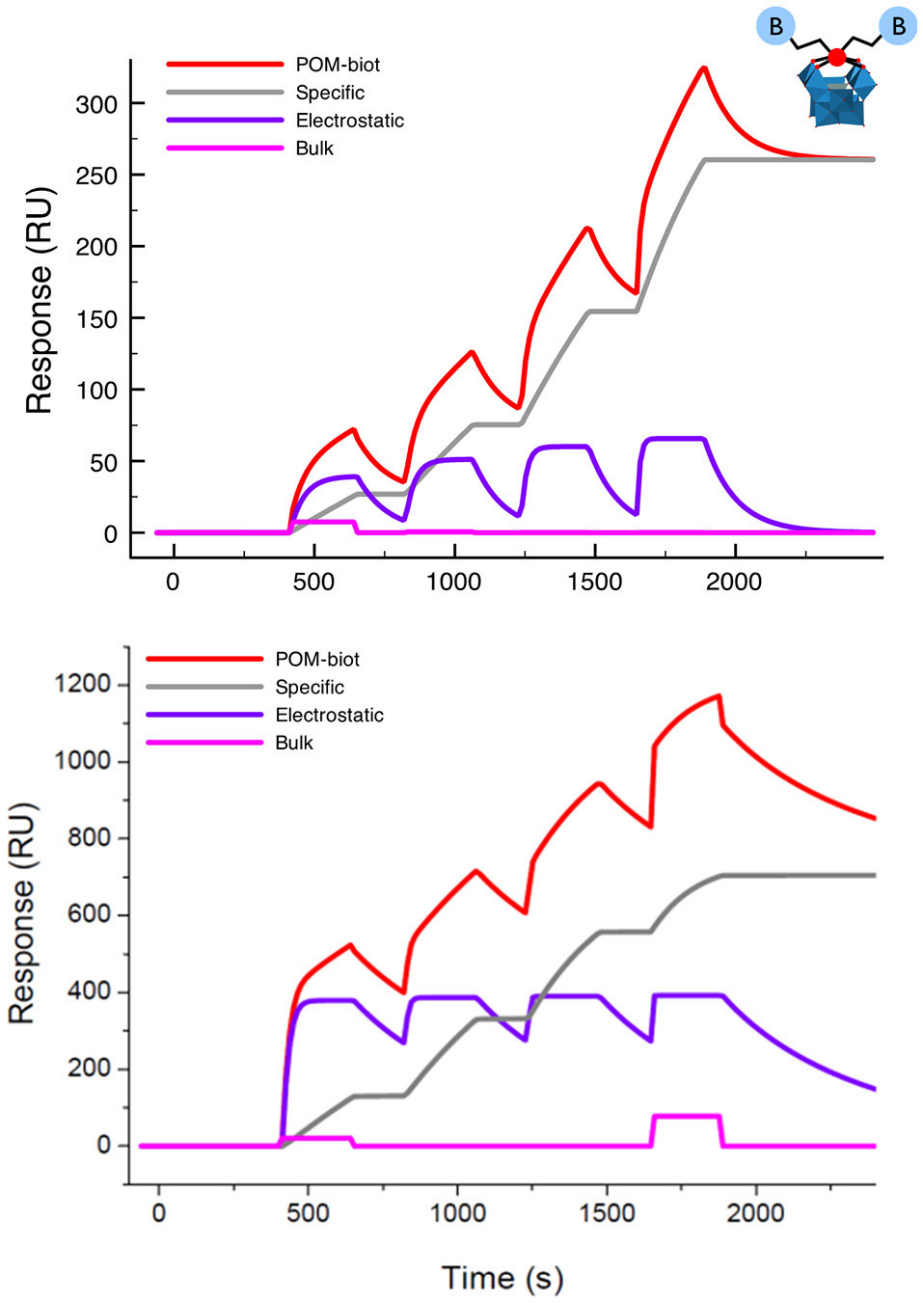
Sensorgram (red lines) obtained for **TBA-POM-biot**_2_ **(Top)** and of **Na-POM-biot**_2_ **(Bottom)** with their corresponding fittings obtained as the result of three concomitant events: a specific binding (gray track), a reversible interaction (purple curve) and a negligible bulk contribution arising from dilution artifacts (pink line). Conditions: flow (10 μl/min) of **POM-biot**_2_ solutions (5, 10, 20, 40 μM) in HBS-ES buffer (pH = 7), with 5% DMSO (only for **TBA-POM-biot**_2_) on an avid functionalized dextran-coated gold chip.

The strength of the interaction between the guests and avidin was then investigated by using the SPR (Surface Plasmon Resonance) technique. To this aim, avidin was immobilized on a dextran-coated gold chip via amide coupling (about 2–4 ng/mm^2^) and exposed to an increasing amount of **TBA-POM-biot**_2_ in HBS-ES buffer containing 5% DMSO, producing the corresponding sensorgram (Figure 3, top). Each injection of **TBA-POM-biot**_2_ produces a clear increase of signal, measured in resonance units (RU), indicating the binding of the compound to the chip surface. After the injections, the flow of the buffered solutions induces a partial dissociation of **TBA-POM-biot**_2_ from the surface but, after four additions, a substantial amount of **TBA-POM-biot**_2_ (250 RU, corresponding to 0.25 ng/mm^2^) remains strongly anchored on the surface.

The sensorgram in Figure 3 is the result of two different biding contributions, featuring different strength and specificity. Indeed, the sensorgram could be successfully fitted with a model accounting for two binding modes. The first is characterized by a slow association, followed by a very slow dissociation rate, resulting in irreversible binding that leads to a stable anchorage of the **TBA-POM-biot**_2_ on the avidin- modified chip.

This behavior is typical of the specific ABC formation, yielding the dissociation constant K_D_ = 2 × 10^−14^ M, which is in the expected range of the avidin-biotin interactions. The second binding mode, which is likely related to unspecific electrostatic interactions, features quite fast association and dissociation rates, resulting in a calculated K_D_ = 4 × 10^−6^ M. Owing to its reversible character, it does not contribute to the final RU counts, i.e., to the amount of **TBA-POM-biot**_2_ that remains irreversibly bound to the avidin-modified chip.

Similar results were obtained for **Na-POM-biot**_2_ (Figure 3, bottom) whereby, taking into account the different loading and the different solvent buffer composition (HBS-ES buffer with no DMSO), the only relevant difference is a lower dissociation constant for the unspecific electrostatic binding (K_D_ = 5 × 10^–8^) M, that is likely ascribed to an easier cationic exchange, in aqueous environment, between the tetracationic avidin subunits and Na^+^.

To evaluate the contribution of the organic groups and of the anionic surface of the POM in the affinity toward avidin, the behavior of other POMs was then screened (Figures S23, S24). Both **TBA-POM-NH**_2_ and **TBA-POM** revealed an unusually high affinity toward avidin, corresponding respectively to a K_D_ of 10^–9^ M and 10^–8^ M, in addition to a reversible contribution with value K_D_ = 10^–6^ M.

The fluorescence quenching profiles and the SPR results indicate that the presence of organic residues, on the POM surface, promotes a stable association with avidin, since both **TBA-POM-NH**_2_ and **TBA-POM** display an improved affinity and on-chip adhesion with respect to totally inorganic species (cfr. **K-POM** in Figure S25). This behavior can be ascribed to a preferential interaction of POM hybrids with the apolar binding site of avidin, thus reinforcing the association (Mock et al., 1988; Rosano et al., 1999).

To highlight the cross-linking potential of the **TBA-POM-biot**_2_, for the organization of multi-avidin networks, we have explored the modification of the SPR response upon alternate addition cycles of the avidin host and of the biotinylated POM guest (Taylor et al., 1991)^1^. Interestingly, addition of further avidin on the chip irreversibly loaded with POM-based ABC in the first experiment cycle, shows a significant increase of the RU values, confirming the additional scavenging of avidin by the **TBA-POM-biot**_2_ (Figure S22), and the occurrence of the bridging binding mode (Figure 1). Moreover, fitting of the SPR curves, yields a K_D_ of ca. 10^–14^ M for the second binding event, that points to an independent behavior of the two biotinylated arms anchored on the POM surface.

**Figure 4:**
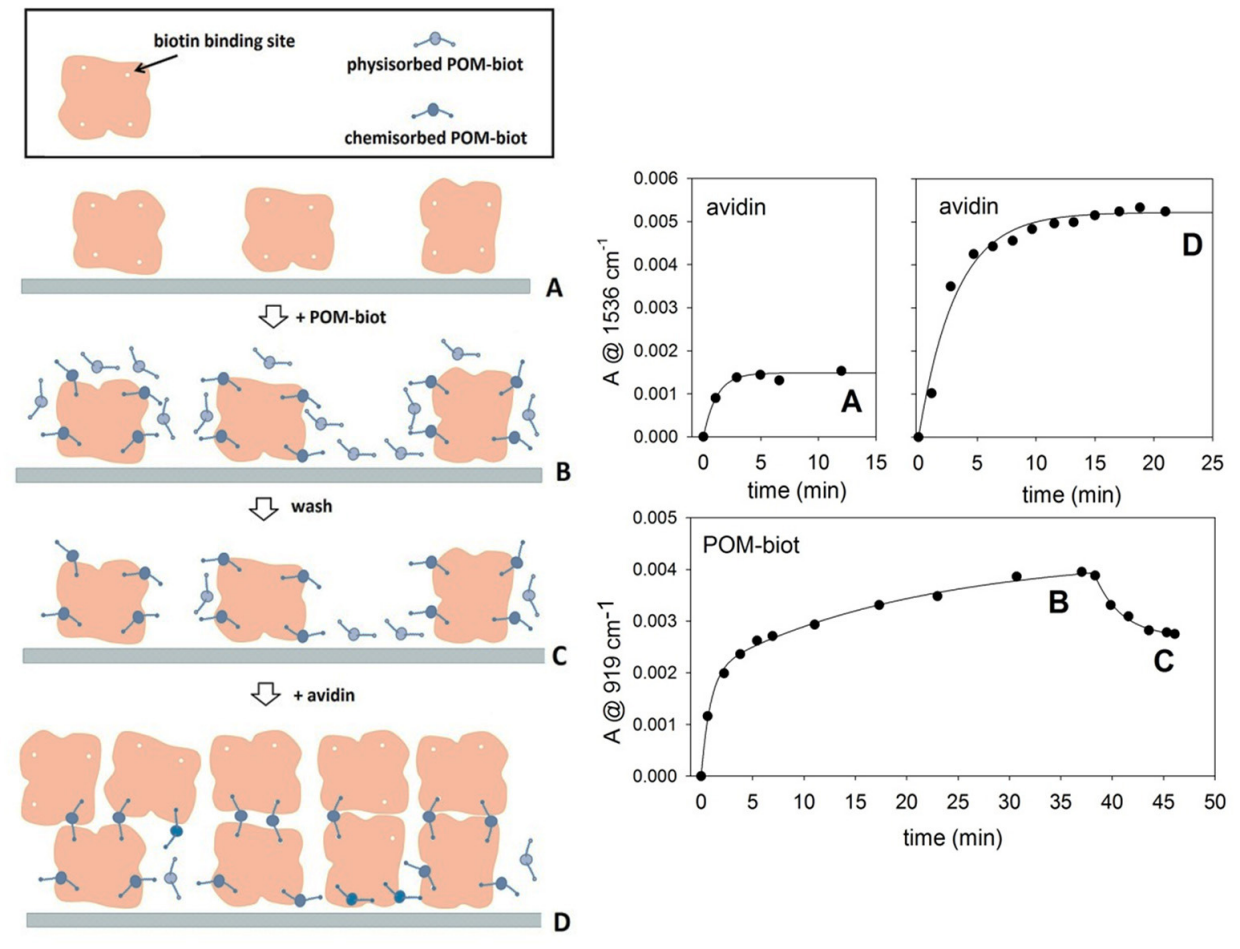
Layer by Layer deposition of alternating avidin/**Na-POM-biot**_2_ layers. The deposition model proposed **(left)** is in agreement with adsorption kinetics of both components **(right)**. The letters A, B, C, D allow to relate the steady infrared signals with the molecular architecture of the films. Marker bands for avidin and POM are at 1,536 and 919 cm^-1^ respectively. Each kinetic curve was obtained in difference mode, thus following the deposition of the last fluxed component.

This POM-directed biotinylated bridge is expected to enable the formation of polymeric structures^2^. The cross-linked interaction between the biotinylated POM and avidin can be carefully controlled by means of a layer-by-layer (LbL) approach (Ariga et al., 2007). ATR-FTIR spectroscopy was thus employed for *in-situ* monitoring of the sequential deposition of alternate **POM-biot**_2_/avidin layers on diamond micro prism which served as internal reflection element and as solid support for the deposited layers (in this case, the sodium salt was used to fully exploit the two binding contributions, while avoiding the competing hydrophobic interactions, see text above). By means of a simple physisorption process, avidin proved to adhere irreversibly onto the bare ATR crystal forming the first protein layer. The intensity of infrared absorption bands, arising from subsequent deposition of alternate POM/avidin layers, reveals that the amount of immobilized avidin increases in presence of a **Na-POM-biot**_2_ layer, according to the deposition process sketched in Figure 4 (steps A-D). The bridging action of **Na-POM-biot**_2_, indeed, promotes the deposition of multiple avidin layers and strengthen the bio-hybrid architecture.

Moreover, *in situ* ATR-FTIR spectroscopy monitoring under flow conditions, shows that the **Na-POM-biot**_2_ adhesion occurs by a strong irreversible host-guest interaction but also via a much weaker binding mode that appears reversible upon rinsing.

The total loading of avidin in the second deposition cycle (step D) is consistent with ca. 3 fold infrared signal enhancement, which is indicative of multiple cross-links directed by the biotinylated POM (Figure 4).

On the other hand, spectra recorded upon addition of **Na-POM-biot**_2_ over a biotin-saturated avidin layer show signal intensities 3 times lower than those obtained with the free avidin, highlighting the role of host-guest interaction in promoting an efficient protein/POM association (Figure S27). A further evidence in this direction has been collected by using streptavidin, another target protein of biotin, which is characterized by a negative surface at neutral pH (pI = 5, Dittmer et al., 1989). In this case, a smaller (25% lower) ATR-FTIR signal can be detected, as a result of the repulsion between the negative charge densities on both surfaces, which hampers the formation of a dense protein layer onto the physisorbed **Na-POM-biot**_2_ layer (Figure S28).

The possibility to access POM based bio-hybrid films is relevant for the design of functional materials with application in sensor technology, electronics, catalysis and nanomedicine (Volatron et al., 2015).

### Catalytic behavior of the POM/avidin assembly

The highly specific interaction resulting from the association of biotin with avidin is commonly exploited for the design of novel semi-synthetic metalloenzymes, whereby the natural protein, functionalized with a biotinylated metal complex, provides a biostructured environment for the catalytic core (Steinreiber and Ward, 2008). Owing to the capability of vacant POMs to activate hydrogen peroxide (Carraro et al., 2006, 2012b; Sartorel et al., 2007), the **Na-POM-biot**_2_**/avidin (2:1)** assembly has been evaluated as potential POM-based bio-hybrid catalyst for the oxygen transfer to a hydrosoluble organic sulfide. The two-step oxidation of L-methionine methyl ester to its corresponding sulfone has thus been considered as model reaction to demonstrate the retention of catalytic properties of the assembled POM (Carraro et al., 2011). The reaction smoothly occurs in buffered aqueous solution [pH 7, at T = 28°C, i.e., under non-denaturating conditions (Thomas et al., 2005; Pordea et al., 2009)] where it was monitored by FT-IR, see Figure S29). Owing to its polymeric nature, **Na-POM-biot**_2_**/avidin** acts as dispersed heterogeneous peroxidase. While the conversion of the L-methionine methyl ester to its sulfoxide is very fast (quantitative conversion was observed in ca. 10 min), further oxidation to sulfone was achieved in ca 24 h, with *t*_1/2_ = 5.1 h (Figure S29). The isolated **Na-POM**-**biot**_2_ displays a similar catalytic activity (*t*_1/2_ = 4.1 h) thus indicating that the avidin ligation is not precluding the access of both substrate and H_2_O_2_ to the POM active sites^3^.

### POM delivery and tracking into HeLa cells

The control of the interaction between POMs and protein can be also exploited to design new delivery strategies. The cell delivery of metal-cores as bio-hybrid conjugates often represents a challenging task, while offering a promising strategy for advanced theranostic and anti-oxidant defense (Orvig and Abrams, 1999; Barry and Sadler, 2013; Albada and Metzler-Nolte, 2016; Liu et al., 2016). The negatively charged surface of the cells, indeed, represents an obstacle to internalization of POMs, that can be considered the molecular analogs of metal-oxide nanoparticles, showing a prominent peroxidase-activity. In addition, tracking of the polyanions often requires disruptive methods, which involves metal detection by X-ray-based spectroscopies. Recently, detection of labeled POMs (Geisberger et al., 2013; Carraro et al., 2014) or encapsulation onto labeled carriers (Geisberger et al., 2011; del Mercato et al., 2014) have been proposed as methods to track hybrid POMs by fluorescence microscopy. We show herein a novel approach, based on a Labeled Streptavidin Biotin (LSAB) complex strategy. POM tracking has thus been investigated by incubating HeLa cells with 0.4 mg/mL of water soluble **Na-POM-biot**_2_ or **Na-POM-NH**_2_, followed by treatment of the pre-incubated cells with Atto 633-labeled Streptavidin, used as a staining agent. As expected, the internalization of the **Na-POM-biot**_2_, that binds strongly to the streptavidin staining agent, is tracked by means of confocal microscopy, thanks to the red-fluorescent streptavidin probe. Vice-versa, because of the weak binding to the biotin-free POM, the labeled streptavidin is readily washed off in the control experiment with **Na-POM-NH**_2._

**Figure 5:**
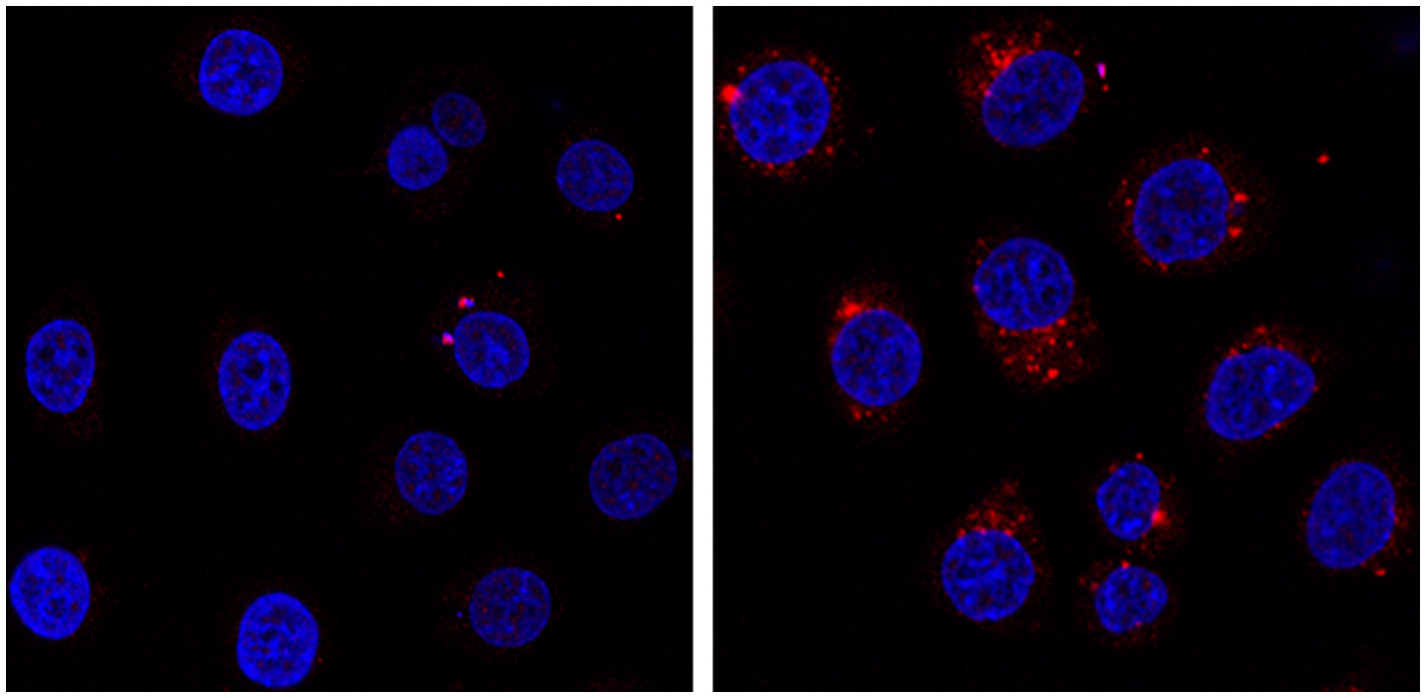
HeLa cells incubated at 37°C for 24 h in presence of 0.4 mg/mL of **Na-POM-NH**_2_ **(Left) Na-POM-biot**_2_ **(Right)**, and stained with Atto 633-Streptavidin (red fluorescent signal). Nucleus stained by Hoechst 33342 (blue). Images acquired by a confocal light scanning microscope (CLSM). A similar signal was seen with a concentration of 0.2 mg/ml POM.

Indeed, the **Na-POM-biot**_2_ incubated cells show well defined red spots in the cytoplasm region (Figure 5 shows nuclei-stained HeLa cells). These defined spots correspond to the intracellular localization of biotin, detected as Labeled Streptavidin Biotin (LSAB) complex. Although this increased biotin content (Dakshinamurti and Chalifour, 1981) is likely ascribed to the biotinylated POM, whose amphiphilic nature promotes the formation of vesicles or aggregates in the physiological cell environment (Geisberger et al., 2013; Fu et al., 2015), further experiments will be required to assess both POM content and its internalization mechanism.

The cytotoxicity of these POMs was determined by flow cytometry, after staining non-viable HeLa cell with propidium iodide (PI), after 24 and 48 h incubation with 60–500 μg/mL of POMs (Figures S30, S31). The experiments showed no decrease of viability and no pro-apoptotic events, suggesting a low cytotoxicity of hybrid POMs even at high doses (Riccardi and Nicoletti, 2006).

## Conclusions

In summary, we have presented for the first time a POM-based bio-conjugate for specific targeting of proteins. The selectivity of **POM-biot**_2_ for avidin was confirmed by CD, fluorimetry, UV-vis titrations, SPR and ATR-FTIR of LbL self-assembly. Among the explored samples, **Na-POM-biot**_2_ displays the highest affinity toward avidin, arising from two distinct binding contributions, i.e., a host-guest specific interaction, strengthened by an unspecific electrostatic interaction, and it allows the cross-linking of proteins to obtain a 2D bio-hybrid network.

This approach provides an efficient engineering of bioactive nano-inorganics and paves the way to a tailored functionalization of the POM surface for bio-recognition, biomimetic catalysis and cell internalization.

## Author contributions

VZ: synthesis, characterization and binding studies of biotinylated POMs. GM: synthesis and characterization of biotinylated POMs. EL: binding studies by SPR. AC: synthesis and characterization of biotinylated POMs as sodium salts. FM: design of binding studies by SPR. LG: LbL approaches, synthesis and characterization of the layers. DM: catalytic studies by FT-IR. LV: design of the LbL approaches. AS and SK: *in vitro* studies with streptavidin. MB and MC: design of the POMs, planning of the experiments, manuscript writing.

### Conflict of interest statement

The authors declare that the research was conducted in the absence of any commercial or financial relationships that could be construed as a potential conflict of interest. The reviewer AA and handling Editor declared their shared affiliation.

## Footnotes

^1^The amount of loaded POM, after a first measurement corresponds to about 10% of the maximum hosting capability by the avidin, meaning that biotin equivalents are in stoichiometric defect.

^2^Transmission electron microscopy (TEM) images obtained for Na-POM-Biot_2_ and avidin (2:1) assembled in aqueous environment show the occurrence of entangled amorphous aggregates with cross section > 50 nm (Figure S26)

^3^Considering the biotin-POM spacer (valeric acid side chain, ca. 13.5 Å) and the POM molecular volume [>600 Å^3^, calculated for Keggin structures, see López et al. (2006)]. The avidin binding pocket [9 Å below the surface of the avidin molecule, with a molecular volume of 293 Å3, see Rosano et al. (1999)] is not expected to encapsulate the POM scaffold, which explains the lack of stereo control registered for the methionine methyl ester sulfoxidation by the bio-hybrid catalyst.
