# Supplementary material for: Selective Targeting of Proteins by Hybrid Polyoxometalates: Interaction Between a Bis-Biotinylated Hybrid Conjugate and Avidin
Source: Front Chem. 2018 Jul 11;6:278. doi: 10.3389/fchem.2018.00278 (PMC6050359; doi:10.3389/fchem.2018.00278)
Supplement: Supplementary file 1 [file Data_Sheet_1.docx]

Supplementary Material

Selective targeting of proteins by hybrid polyoxometalates: interaction between a bis-biotinylated hybrid conjugate and avidin

**V. A. Zamolo,^a^ G. Modugno, ^a^ E. Lubian, ^a^ A. Cazzolaro, ^a^ F. Mancin, ^a^ L. Giotta,^b^ D. Mastrogiacomo,^b^ L. Valli,^b^ A. Saccani,^c^ S. Krol,^c,d^ M. Bonchio,*^a^ M. Carraro*^a^**

**Correspondence:** Corresponding Author: **mauro.carraro@unipd.it, marcella.bonchio@unipd.it**

# Supplementary Data

**Materials**

Chemicals and solvents were purchased from Sigma Aldrich (Milan, Italy) and used as received.

**Instrumentation**

1H and 13C NMR spectra were recorded with Bruker AV300 instruments operating at 300.13 MHz or at 75.4 MHz, respectively, Si(CH3)4 was used as reference.

183W and 29Si NMR spectra were recorded with a Bruker Avance DRX 400 instrument operating at 16.67 and 79.50 MHz, respectively, using Na2WO4 in D2O and Si(CH3)4 in CDCl3 as external references.

FTIR (KBr) spectra were collected with a Thermo Quest Nicolet 5700 instrument.

ESI-MS spectra were obtained with an Agilent LC/MSD Trap SL spectrometer, by using a capillary potential of 1500 V.

Fluorimetric analyses were performed with a Perkin–Elmer LS50B instrument with 1 cm quartz cell irradiation at 280 nm.

**Catalysis details**

The ATR-FTIR spectroscopy was employed for following the oxidation of methionine methyl ester by H_2_O_2_ in PBS buffer at pH 7.0. L-methionine methyl ester concentration was 85 mM. Both free **Na-POM-biot_2_** and avidin-bound **Na-POM-biot_2_** were tested as catalysts at the concentration of 70 µM. In the latter case, avidin was added in a 1:2 molar ratio with respect **Na-POM-biot_2_**. The reaction was triggered by the addition of an excess of hydrogen peroxide (final concentration 200 mM). ATR-FTIR spectra of aliquots of the reaction mixture, withdrawn at different times, were acquired with a Perkin Elmer Spectrum One spectrometer, equipped with an ATR horizontal sampling apparatus (Smith Detection, USA, former SensIR technologies). The IRE was a three-bounce diamond microprism. The spectra were acquired at a 4 cm^-1^ resolution against a methionine methyl ester 85 mM blank. Only absorbance changes arising from the production of oxidation products (sulfoxide and sulfone) were detected in the infrared spectrum. Difference signals intensities were plotted versus time and the plots were fitted with a first order kinetics law. The marker band for the sulfoxide was at 1008 cm^-1^, while the marker bands for the sulfone were at 1237 cm^-1^ and 1133 cm^-1^. Sulfone formation was not observed for a blank reaction without POM, whilst sulfoxide production was found slower.

**Cell Viability – FACS analysis.**

Non transfected or modified HeLa cell (purchased from ATCC (CRL-5802)) were incubated with **Na-POM-NH_2_** or **Na-POM-biot_2_** in a concentration range of 0.06-0.5 mg/ml as described above. Cells without POMs were used as control. After 24 and 48 h incubation, 5 x 10^5^ cell were collected by trypsinization and washed with PBS containing 1% BSA. The staining of nonviable cells was performed with propidium iodide (PI) (50 µg/mL in PBS) for 5 minutes at room temperature. The samples were analyzed immediately on a flow cytometer (FACSCanto II, BD Biosciences) with excitation of PI at 488 nm.

# Supplementary Figures

**Supplementary Scheme S1:** Synthesis of **TBA-POM-biot_2_**


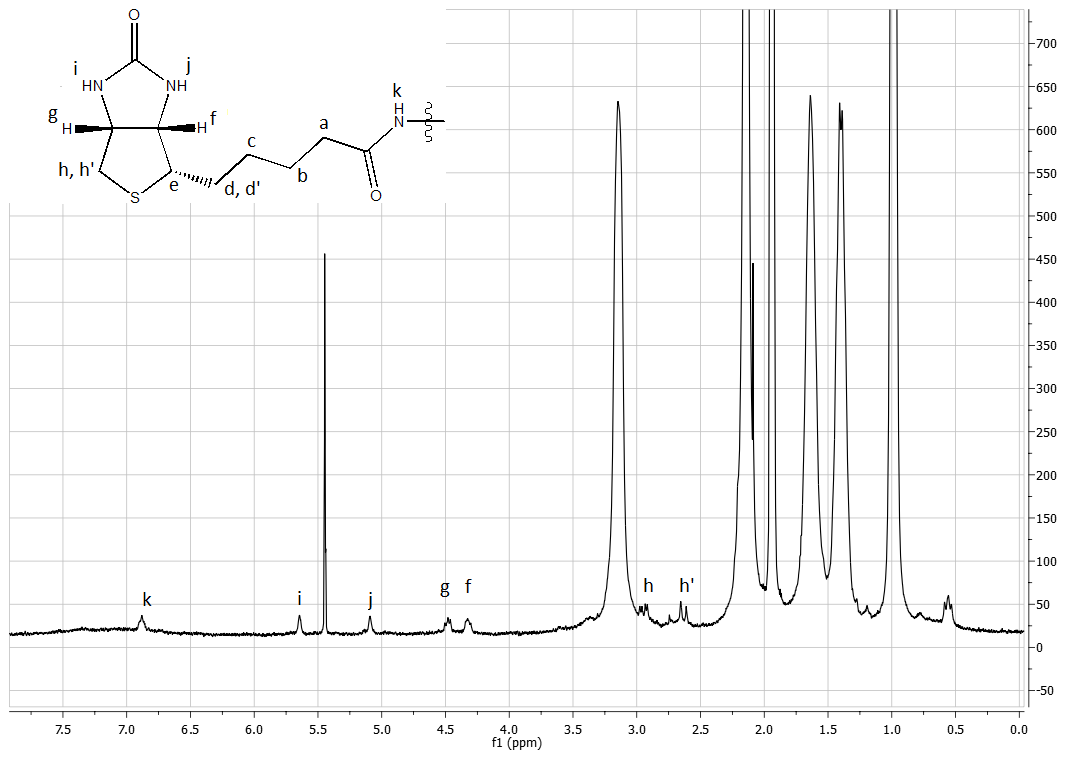


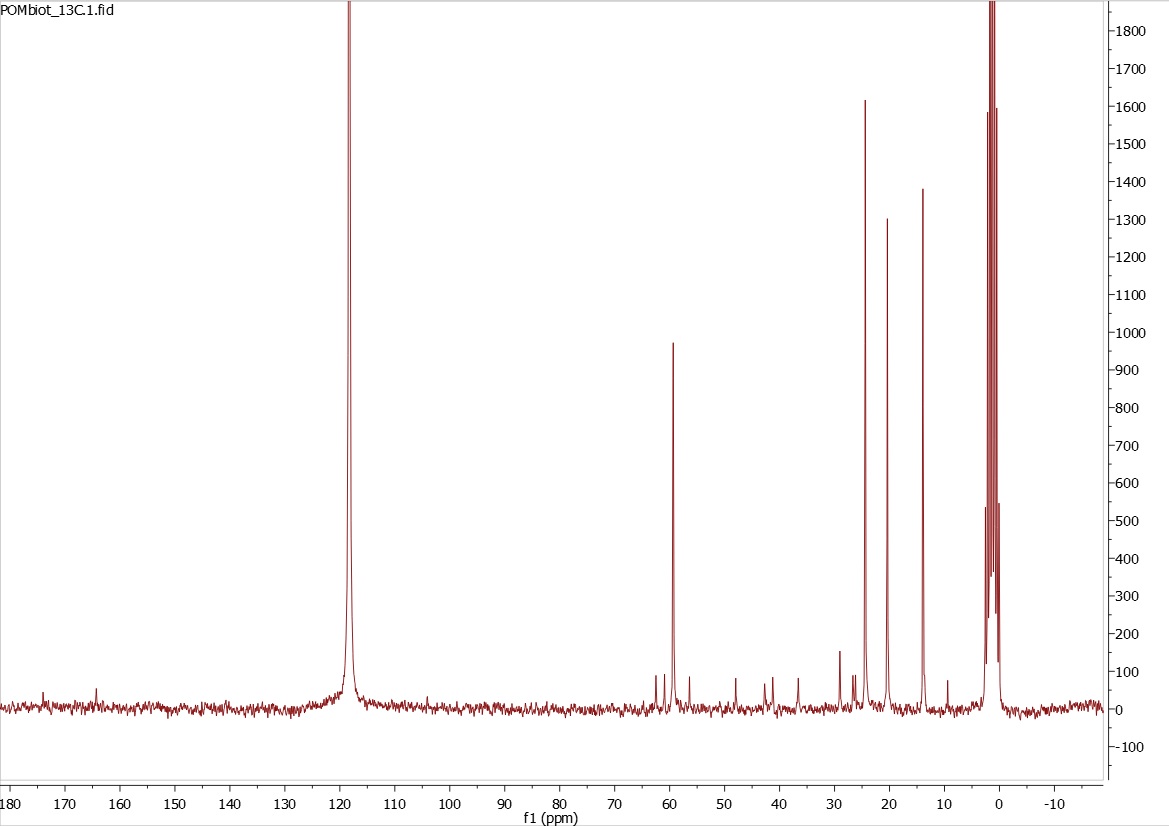
 **Supplementary Figure S1:** ^1^H NMR (CD_3_CN) of **TBA-POM-Biot_2_**.

**Supplementary Figure S2:** ^13^C NMR (CD_3_CN) of **TBA-POM-Biot_2_**.


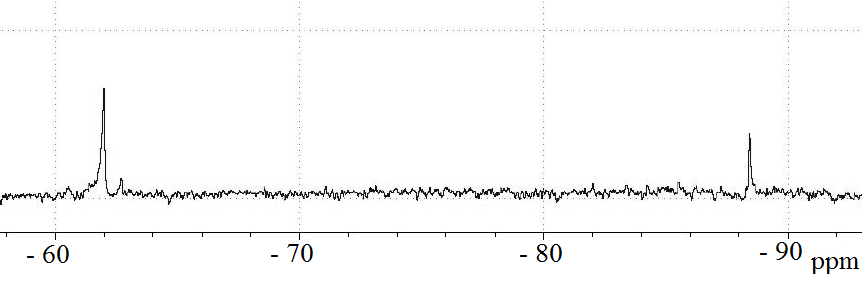


**Supplementary Figure S3:** ^29^Si NMR (CD_3_CN) spectrum of **TBA-POM-Biot_2_**.


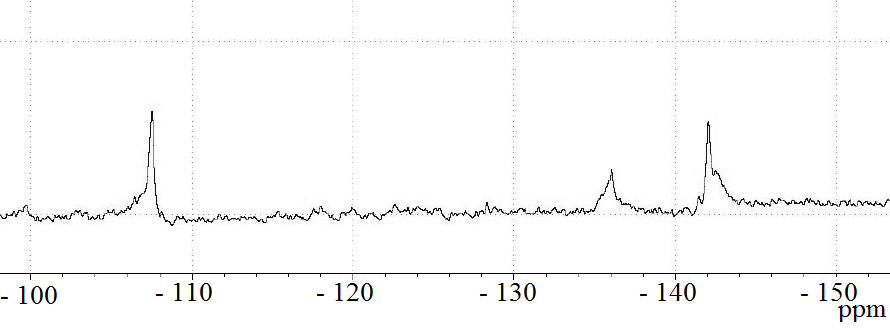


**Supplementary Figure S4:** ^183^W NMR (CD_3_CN) spectrum of **TBA-POM-Biot_2_**.

***
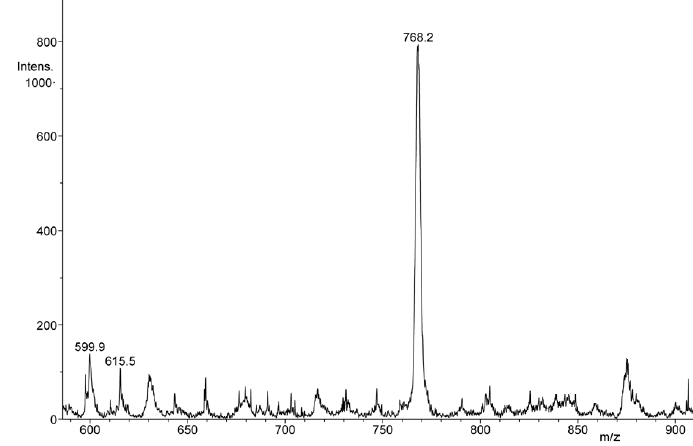
***

**Supplementary Figure S5:** ESI-MS ((-), CH_3_CN) of **TBA-POM-Biot_2_**.


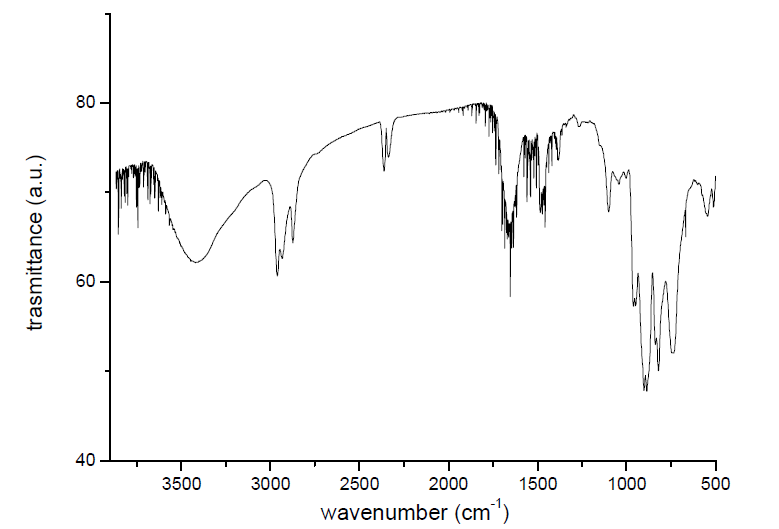


Transmittance

**Supplementary Figure S6:** FT-IR (KBr) of **TBA-POM-Biot_2_**.


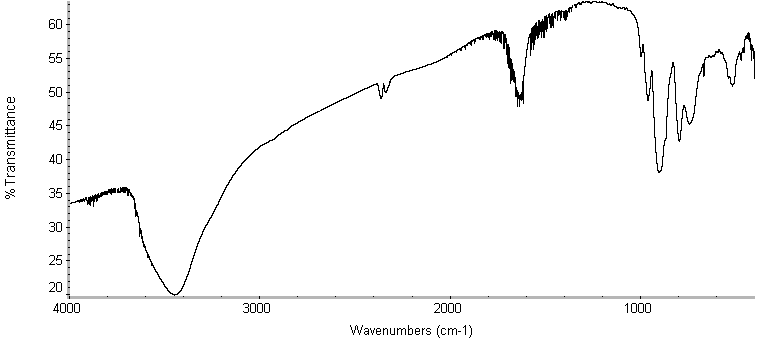

(a)

(b)

**Supplementary Figure S7:** FT-IR (KBr) of (a) **Na-POM-NH_2_** and (b) **Na-POM-Biot_2_**

**Supplementary Figure S8** Dichroic spectrum of avidin (1x10^-6^ M, in PBS, 25°C) in the presence of increasing equivalents of biotin dissolved in PBS.


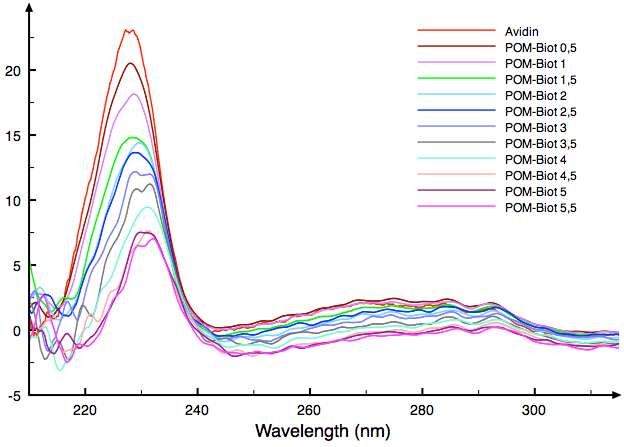


**Supplementary Figure S9** Dichroic spectrum of avidin (1x10^-6^ M, in PBS, 25°C) in the presence of increasing amount of **TBA-POM-Biot_2_** dissolved in PBS.

**Supplementary Figure S10** Dichroic spectrum of avidin (1x10^-6^ M, in PBS, 25°C) in the presence of increasing amount of **TBA-POM** dissolved in PBS.


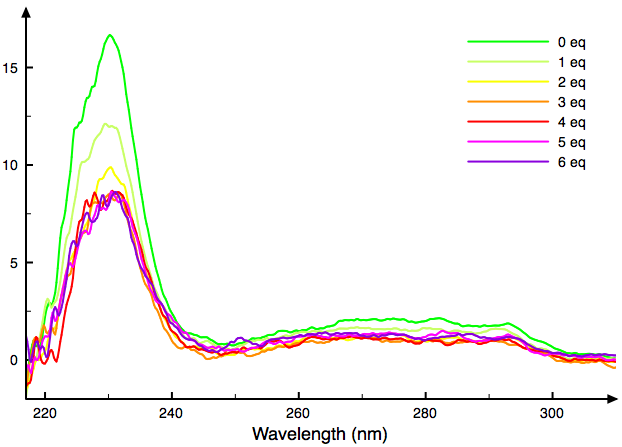


**Supplementary Figure S11** Dichroic spectrum of avidin (1x10^-6^ M, in PBS, 25°C) in the presence of increasing amount of **K-POM** dissolved in PBS.

**Supplementary Figure S12:** Fluorescence spectra of avidin (1x10^-6^ M, in PBS, 25°C) in the presence of increasing amount of biotin dissolved in PBS. K_sv_=3.0x10^5^ M^-1^

**Supplementary Figure S13:** Fluorescence spectra of avidin (1x10^-6^ M, in PBS with 5% DMSO, 25°C) in the presence of increasing amount of **TBA-POM-Biot_2_** dissolved in PBS with5% DMSO. K_sv_=9.6x10^5^ M^-1^

**Supplementary Figure S14:** Fluorescence spectra of avidin (1x10^-6^ M, in PBS with 5% DMSO, 25°C) in the presence of increasing equivalents of **POM-NH_2_** dissolved in PBS 5% DMSO. Inset: K_SV_=3.0x10^5^ M^-1^

**Supplementary Figure S15:** Fluorescence spectra of avidin (1x10^-6^ M, in PBS with 5% DMSO, 25°C) in the presence of increasing amount of **TBA**-**POM** dissolved in PBS 5% DMSO. Inset: K_SV_=3.1x10^5^ M^-1^

**Supplementary Figure S16:** Fluorescence spectra of avidin (1x10^-6^ M, in PBS 5% DMSO, 25°C) in the presence of increasing equivalents of K-**POM** dissolved in PBS with 5% DMSO. Inset: K_SV_=1,1x10^5^ M^-1^

**
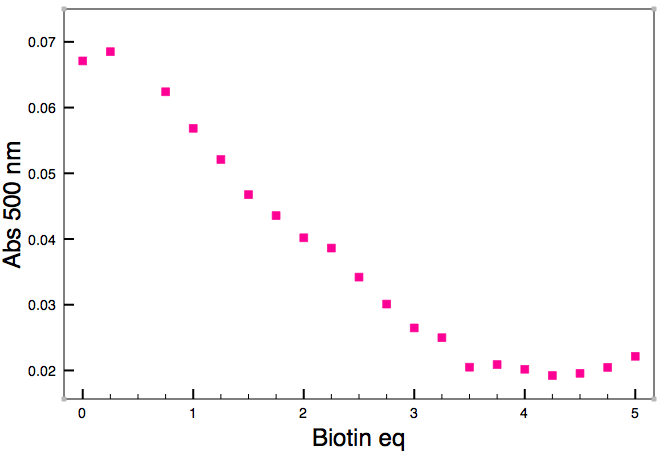
**

**Supplementary Figure S17.** UV-VIS spectra of tetrameric avidin-HABA (0.65x10^-6^ M, in HEPES buffer, 25°C) in the presence of increasing amount of biotin equivalents.

**
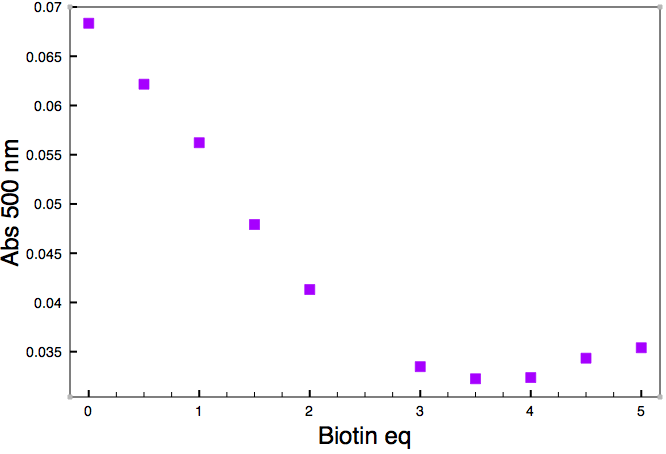
**

**Supplementary Figure S18.** UV-VIS spectrum of tetrameric avidin-HABA (0.65x10^-6^ M, in HEPES buffer with 5% DMSO, 25°C) in the presence of increasing equivalents of **TBA-POM-Biot_2_** dissolved in HEPES buffer 5% DMSO. *Deviations are due to the scattering effect, observed above 3 equivalents of biotin (corresponding to 1.5* ***TBA-POM-Biot_2_*** *equivalents), arising from the decreased solubility of the adduct.*


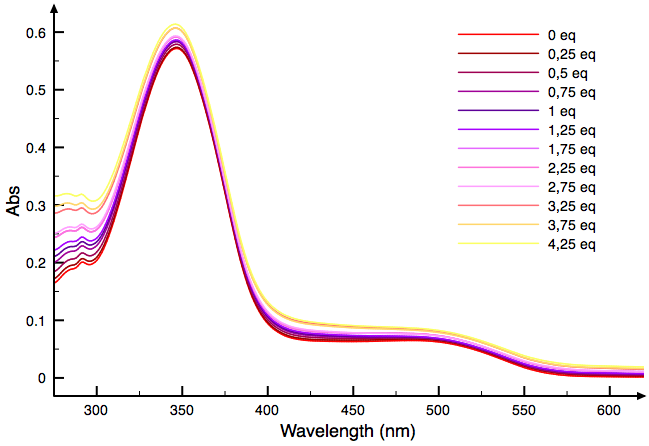


**Supplementary Figure S19** UV-VIS spectrum of tetrameric avidin-HABA (0.65x10^-6^ M, in HEPES buffer with 5% DMSO, 25°C) in the presence of increasing equivalents of **TBA-POM-NH_2_** dissolved in HEPES buffer 5% DMSO.

**Supplementary Figure S20.** UV-VIS spectrum of tetrameric avidin-HABA (0.65x10^-6^ M, in HEPES buffer with 5% DMSO, 25°C) in the presence of increasing equivalents of **TBA-POM** dissolved in HEPES buffer with 5% DMSO.

**Supplementary Figure S21** UV-VIS spectrum of tetrameric avidin-HABA (0,65x10^-6^ M, in HEPES buffer with 5% DMSO, 25°C) in the presence of 3 equivalents of biotin and increasing amount of **TBA-POM-Biot_2_** equivalents diluted in HEPES buffer 5% DMSO. Correspondent equivalents of biotin are reported in brackets.

**SPR measurements**

**Supplementary Figure S22** Fitted sensorgram (black line) obtained after subsequent injections of **Avidin** (5 μM, 10 μM, 20 μM, 40 μM) in HBS-ES buffer. The specific contribution corresponds to the pink track, the electrostatic process to the blue one. The violet line is the bulk interference.

**Supplementary Figure S23** Fitted sensorgram (black line) obtained after subsequent injections of **TBA-POM-NH_2_** (5 μM, 10 μM, 20 μM, 40 μM) in HBS-ES buffer with 5% DMSO. The specific contribution corresponds to the pink track, the electrostatic process to the blue one.

**Supplementary Figure S24** Fitted sensorgram (black line) obtained after subsequent injections of **TBA-POM** (1 μM, 2 μM, 3 μM, 5 μM, 10 μM) in HBS-ES buffer with 5% DMSO. The specific contribution corresponds to the pink track, the electrostatic process to the blue one.

**Supplementary Figure S25** Fitted sensorgram (black line) obtained after subsequent injections of **K-POM** (5 μM, 10 μM, 20 μM, 40 μM) in HBS-ES buffer. Since in this case no functional groups are present, the events contributing to the final shape are very similar and purely electrostatic.

**
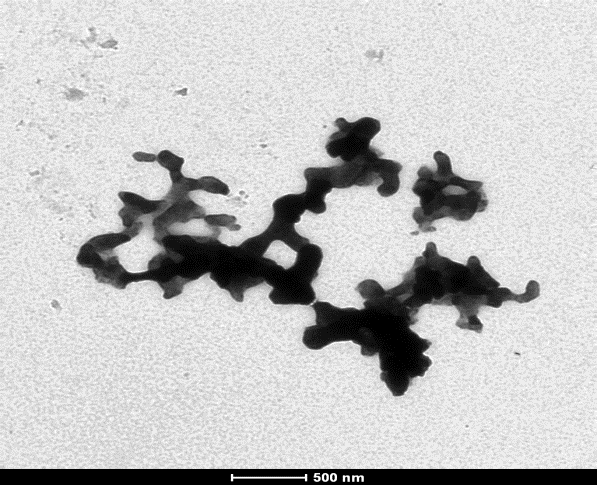
**

**
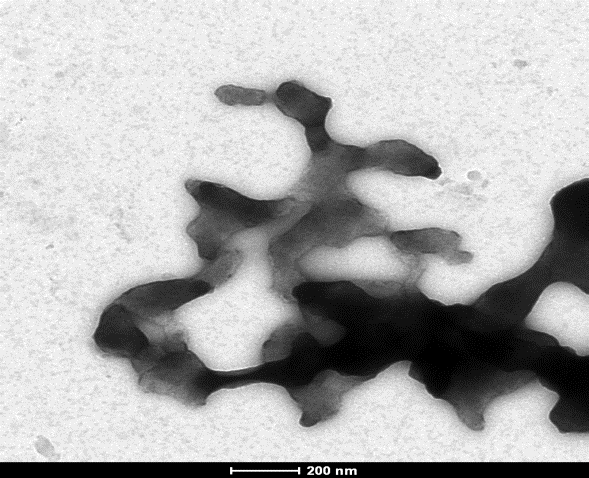
**

**Supplementary Figure S26:** TEM image of **Na-POM-Biot_2_** with avidin, drop casted from a aqueous solution with 5% DMSO.

**Supplementary Figure S27** Deposition kinetics of **Na-POM-biot_2_** on the surface of the IRE coated with avidin (top) and avidin/biotin (bottom). Avidin and biotin-saturated avidin were immobilized on the diamond IRE by physisorption. The absorption at 919 cm^-1^, whose intensity is monitored during the perfusion process, represents the marker band for **Na-POM-biot_2_.**

**

**

**Supplementary Figure S28 ATR-FTIR** spectra collected for: A) physisorbed **Na-POM-biot_2_** layer; B): Avidin immobilized onto a **Na-POM-biot_2_** layer; C) Streptavidin immobilized onto a **Na-POM-biot_2_** layer; D) Biotin-saturated avidin immobilized onto a **Na-POM-biot_2_** layer. Protein concentration: 0.01 mg/mL in PBS buffer at pH7. For protein spectra acquisition, the difference mode was adopted taking the spectrum A as background.


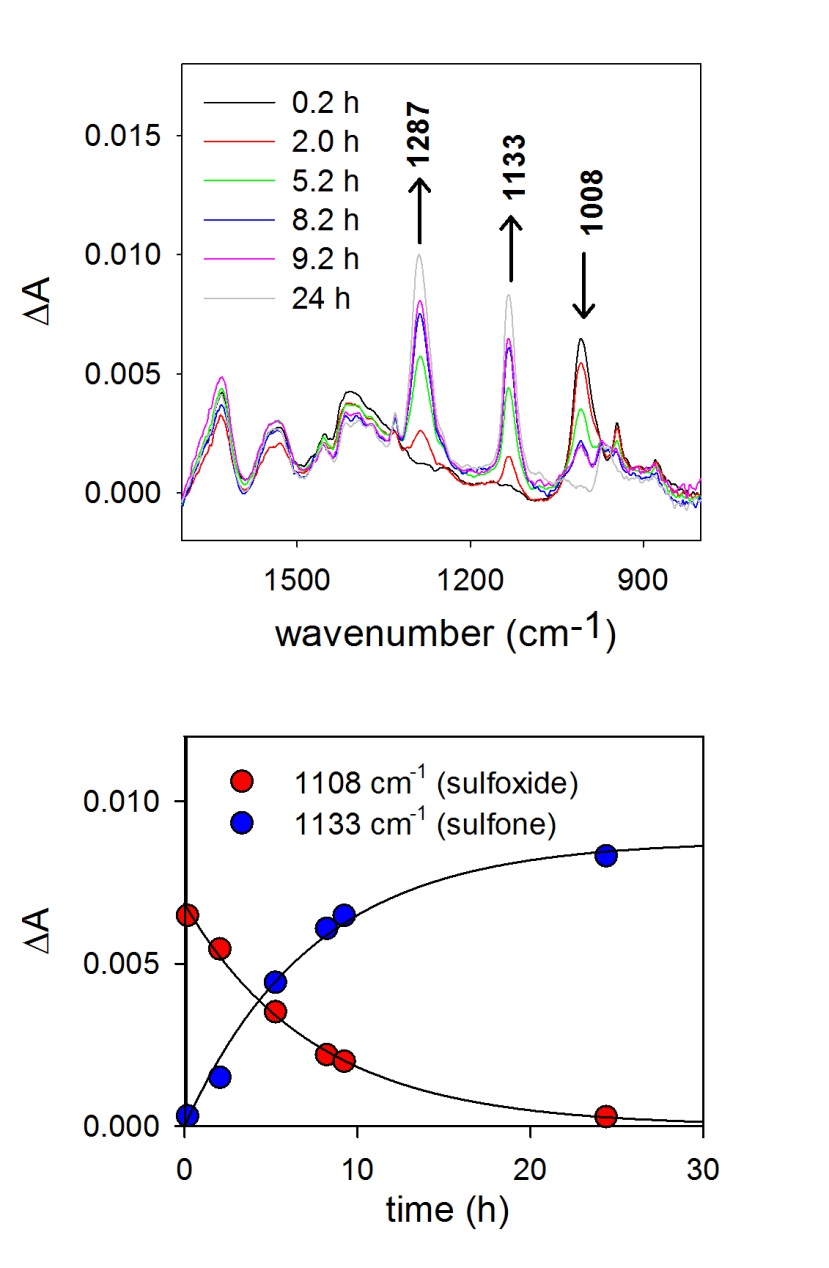


Supplementary Figure S29 ATR-FTIR spectra evolution and kinetic traces for the oxidation of methionine methylester (85 mM) by H_2_O_2_ (200 mM) in PBS buffer at pH 7.0 in the presence of Na-POM-biot_2_ / avidin (70 µM).

**Supplementary Figure S30.** Viability of HeLa cells by propidium iodide staining for dead cells incubated with different doses (mg/mL) of POMs, at 24h (top) and 48h (bottom).

**
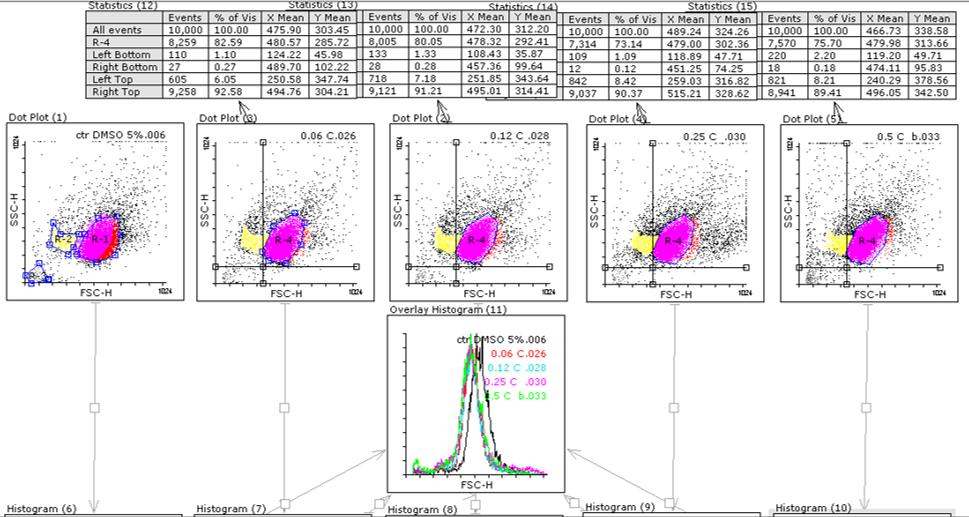
**

**
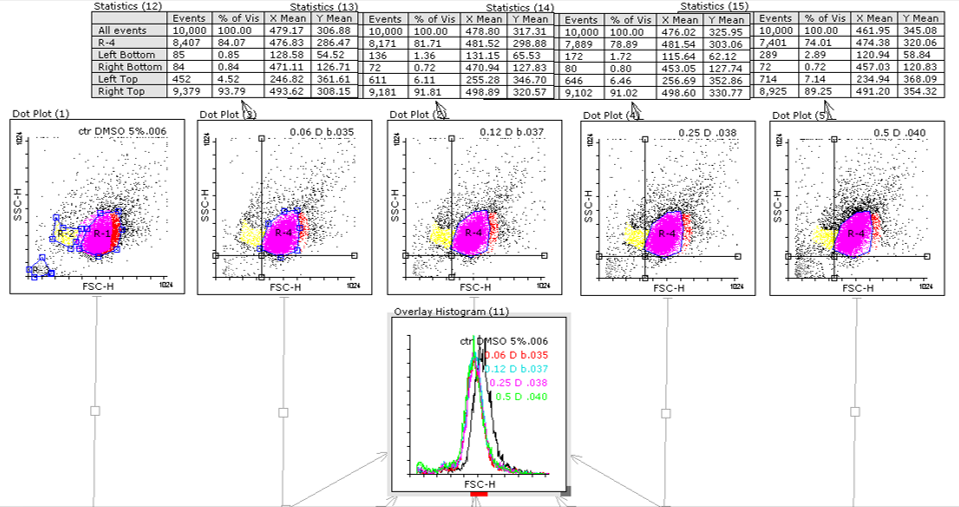
**

**Supplementary Figure S31.** Flow Cytometry (Fluorescence-activated cell sorting, FACS) of HeLa cells incubated with different doses (mg/mL) of **Na-POM-Biot_2_** (top) and **Na-POM-NH_2_** (bottom).
